# A Deep Artificial Neural Network−Based Model for Prediction of Underlying Cause of Death From Death Certificates: Algorithm Development and Validation

**DOI:** 10.2196/17125

**Published:** 2020-04-28

**Authors:** Louis Falissard, Claire Morgand, Sylvie Roussel, Claire Imbaud, Walid Ghosn, Karim Bounebache, Grégoire Rey

**Affiliations:** 1 Inserm (Institut National de la Santé et de la Recherche Médicale) - CépiDc (Centre d'epidémiologie sur les causes médicales de Décès) Le Kremlin Bicêtre France; 2 Université Paris Saclay Le Kremlin Bicêtre France

**Keywords:** machine learning, deep learning, mortality statistics, underlying cause of death

## Abstract

**Background:**

Coding of underlying causes of death from death certificates is a process that is nowadays undertaken mostly by humans with potential assistance from expert systems, such as the Iris software. It is, consequently, an expensive process that can, in addition, suffer from geospatial discrepancies, thus severely impairing the comparability of death statistics at the international level. The recent advances in artificial intelligence, specifically the rise of deep learning methods, has enabled computers to make efficient decisions on a number of complex problems that were typically considered out of reach without human assistance; they require a considerable amount of data to learn from, which is typically their main limiting factor. However, the CépiDc (Centre d’épidémiologie sur les causes médicales de Décès) stores an exhaustive database of death certificates at the French national scale, amounting to several millions of training examples available for the machine learning practitioner.

**Objective:**

This article investigates the application of deep neural network methods to coding underlying causes of death.

**Methods:**

The investigated dataset was based on data contained from every French death certificate from 2000 to 2015, containing information such as the subject’s age and gender, as well as the chain of events leading to his or her death, for a total of around 8 million observations. The task of automatically coding the subject’s underlying cause of death was then formulated as a predictive modelling problem. A deep neural network−based model was then designed and fit to the dataset. Its error rate was then assessed on an exterior test dataset and compared to the current state-of-the-art (ie, the Iris software). Statistical significance of the proposed approach’s superiority was assessed via bootstrap.

**Results:**

The proposed approach resulted in a test accuracy of 97.8% (95% CI 97.7-97.9), which constitutes a significant improvement over the current state-of-the-art and its accuracy of 74.5% (95% CI 74.0-75.0) assessed on the same test example. Such an improvement opens up a whole field of new applications, from nosologist-level batch-automated coding to international and temporal harmonization of cause of death statistics. A typical example of such an application is demonstrated by recoding French overdose-related deaths from 2000 to 2010.

**Conclusions:**

This article shows that deep artificial neural networks are perfectly suited to the analysis of electronic health records and can learn a complex set of medical rules directly from voluminous datasets, without any explicit prior knowledge. Although not entirely free from mistakes, the derived algorithm constitutes a powerful decision-making tool that is able to handle structured medical data with an unprecedented performance. We strongly believe that the methods developed in this article are highly reusable in a variety of settings related to epidemiology, biostatistics, and the medical sciences in general.

## Introduction

The availability of up-to-date, reliable mortality statistics is a matter of significant importance in public health−related disciplines. As an example, the monitoring of leading causes of deaths is an important tool for public health practitioners and has a considerable impact on health policy−related decision-making processes [1-6]. The collection of said data, however, is complex, time-consuming, and usually involves the coordination of many different actors, starting from medical practitioners writing death certificates following an individual’s passing, to the finalized mortality statistics’ diffusion by public institutions. One example of a nontrivial task involved in this process is the identification of the underlying cause of death from the chain of events reported by the medical practitioner in the death certificate [7]. According to the International Statistical Classification of Diseases and Related Health Problems, the underlying cause of death is defined as “(a) the disease or injury which initiated the train of morbid events leading directly to death, or (b) the circumstances of the accident or violence which produced the fatal injury” [8]. As underlying causes of death are the main information used in the tabulation of mortality statistics, extracting them from death certificates is of paramount importance.

Nowadays, in order to preserve spatial and temporal comparability, the underlying cause of death is usually identified from an expert system [9], such as the Iris software (The Iris Institute) [10], a form of artificial intelligence that encodes a series of World Health Organization (WHO)−defined coding rules as an entirely hand-built knowledge base stored in *decision tables* [10]. Unfortunately, these decision systems fail to handle a significant amount of more complex death scenarios, typically including multiple morbidities or disease interactions. These cases then require human evaluation, consequently leading to a time-consuming coding process, potentially subject to distributional shift across both countries and years, sensibly impairing the statistics’ comparability.

In the past few years, the field of artificial intelligence has been subject to a significant expansion, mostly led by the recent successes encountered in the application of deep artificial neural network−based predictive models in various tasks, such as image analysis, voice analysis, or natural language processing. These methods have been known to outperform expert systems but usually require vast amounts of data on which to train to do so, which is oftentimes prohibitive. On the other hand, a number of countries, including France, have been storing their death certificates, along with their derived underlying causes, in massive databases, thus providing an optimal setting to use deep learning methods.

The following article formulates the process of extracting the underlying cause of death from death certificates as a statistical predictive modelling problem and proposes to solve it with a deep artificial neural network. The following section focuses on describing the structured information contained in a death certificate. The Methods section introduces the neural network architecture used for the task of predicting the underlying cause of death. The Results section reports the performances obtained from training the neural network on French death certificates from 2000 to 2015—about 8 million training examples—as well as a comparison with prediction performances obtained using the Iris software, the current state-of-the-art for this predictive task and solution used in numerous countries for underlying cause of death coding. Finally, the Practical Application section showcases the potential use of the presented approach in epidemiology with a focus on opioid overdose−related deaths in France.

## Methods

### Dataset

The dataset used during this study consists of every available death certificate found in the CépiDc (Centre d’épidémiologie sur les causes médicales de Décès) database from 2000 to 2015 and their associated cause of death, coded either by human experts or the Iris software depending on the certificate’s complexity. The entire dataset represents over 8 million training examples and records various information about their subjects, with varying predictive power with regard to the underlying cause of death. This article aims to derive a deep neural network−based predictive model explaining the underlying cause of death from the information contained within death certificates by solving the following modelling problem:

*P*(*UCD*|*DC*) = *ƒ*(*DC*) (1)

with *DC* representing the information contained in a French death certificate, *UCD* representing its corresponding underlying cause of death, and *ƒ* representing a neural network−based predictive function.

In order to model the underlying cause of death from this information, the following items were selected as explanatory variables: (1) the causal chain of events leading to death, (2) age, (3) gender, and (4) year of death.

### Causal Chain of Death

**Figure 1:**
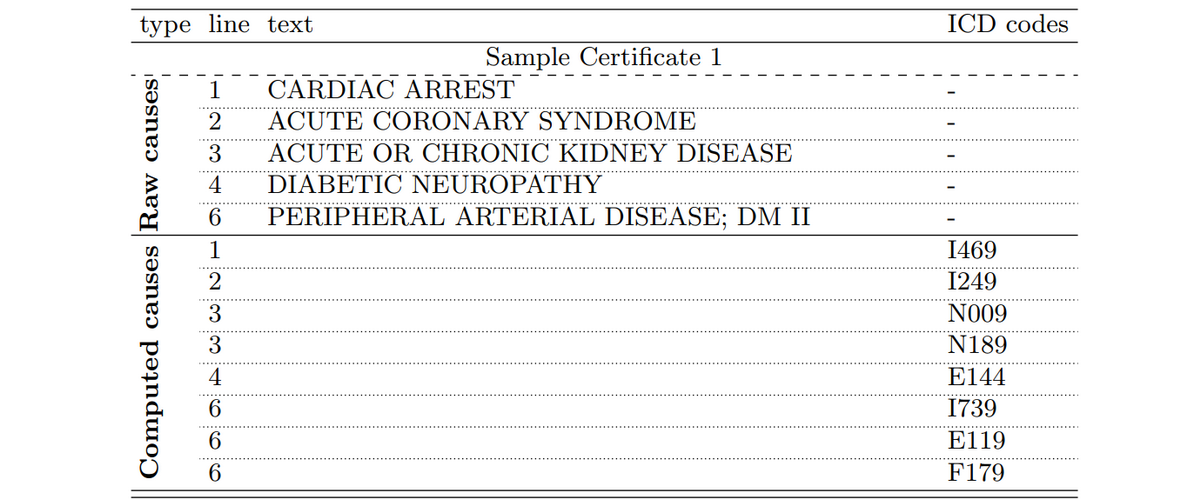
Example of causal chain of death as found on a French death certificate. Its corresponding underlying cause of death was defined as “diabetes mellitus type 2 [DM II], with multiple complications." ICD: International Statistical Classification of Diseases and Related Health Problems.

The causal chain of death constitutes the main source of information available on a death certificate in order to devise its corresponding underlying cause of death. It typically sums up the sequence of events that led to the subject’s death, starting from immediate causes, such as cardiac arrest, and progressively expanding into the individual’s past to the underlying causes of death (see Figure 1). The latter being the target of the investigated predictive model, the information contained in the causal chain of death is of paramount importance to the decision process leading to the underlying cause of death’s establishment. In order to enforce the comparability of death statistics across countries, the coding of the underlying cause of death from the causal chain of events is defined from a number of WHO-issued rules, oftentimes reaching casuistry on more complex situations [11].

The WHO provides countries with a standardized causal chain of events format, which France, alongside every country using the Iris software, follows. This WHO standard asks of the medical practitioner in charge of reporting the events leading to the subject’s passing to fill out a two-part form in natural language. The first part is comprised of four lines, in which the practitioner is asked to report the chain of events, from immediate to underlying cause, in inverse causal order (ie, immediate causes are reported on the first lines and underlying causes on the last lines). Although four lines are available for reporting, they need not all be filled. In fact, the last available lines are rarely used by the practitioner (eg, line four was used less than 20% of the time in the investigated dataset).The second part is comprised of two lines in which the practitioner is asked to report any “other significant conditions contributing to death but not related to the disease or condition causing it” [12] that the subject may have been suffering from. Although this part might seem at first sight to have close to no impact on the underlying cause of death, some coding rules ask that the latter should be taken from this part of the death certificate. As an example, the underlying cause of death of an individual with AIDS who died from Kaposi’s sarcoma should be coded as AIDS, although this condition might be considered by the medical practitioner as a comorbidity and, as such, written on the certificate’s second part. Consequently, this part of the death certificate also presents some vital information for the investigated predictive model and, as such, should be included as input variable.

In order to counter the language-dependent variability of death certificates across countries, a preprocessing step is typically applied to the causal chain of events leading to the individual’s death, where each natural language−based line on the certificate is converted into a sequence of codes defined by the 10th revision of the International Statistical Classification of Diseases and Related Health Problems (ICD-10). The ICD-10 is a medical classification defined by the WHO [8] defining 14,199 medical entities [13] (eg, diseases, signs and symptoms, among others) distributed over 22 chapters and encoded with three or four alpha decimal symbols (ie, one letter and 2 or 3 digits), 7404 of which are present in the investigated dataset. The WHO-defined decision rules governing the underlying cause of death process are actually defined from this ICD-10−converted causal chain, and the former is to be reported as a unique ICD-10 code.

**Figure 2:**
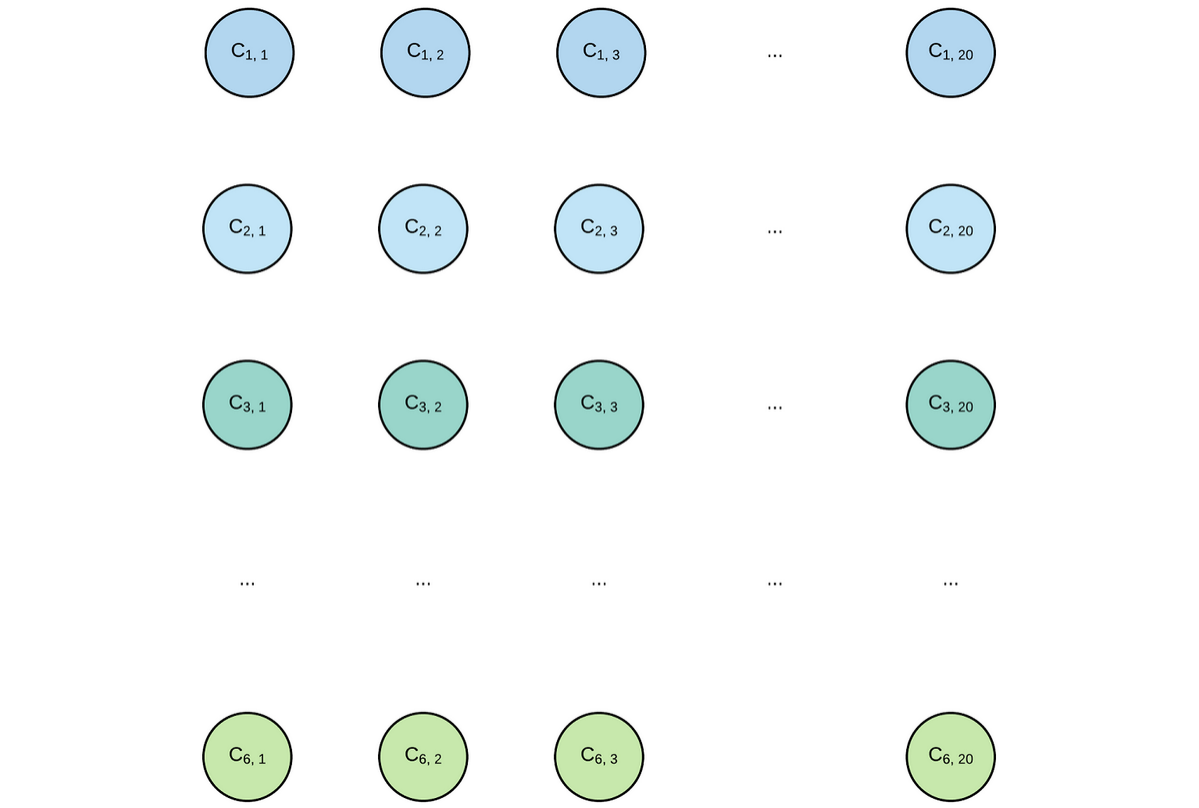
Causal chain of death encoded as a 3D tensor. Each node represents an ICD-10 code as a 7404-dimensional dummy variable. Its row and column positions respectively denote the corresponding code’s line and rank in the corresponding certificate. Ci,j: denotes the jth code at the ith line in the death certificate; ICD-10: 10th revision of the International Statistical Classification of Diseases and Related Health Problems.

The processed causal chain of death, in its encoded format, can be assimilated as a sequence of six varying-length sequences of ICD-10 codes. In order to simplify both the model and computations, this hierarchical data structure will hereon be assimilated, as seen in Figure 2, as a padded 6-by-20 grid of ICD-10 codes, with rows and columns denoting a code’s line and rank in line, respectively; 20 is the maximal number of ICD-10 codes found on a causal chain line in all certificates present in the investigated dataset. Several more subtle approaches to this grid-like assimilation were explored prior to the experiment reported in this article, but all yielded models with significantly inferior predictive power. Although this encoding scheme apparently prevents the encoding to handle death certificates with at least one line containing more than 20 codes, the model introduced further sees no such limitation. Bigger certificates can be processed without trouble with an appropriately larger code matrix encoding, with theoretically no significant loss in performance, since the model is translation invariant [14].

The question of encoding ICD-10 codes in a statistically exploitable format is another challenge in itself. A straightforward approach would be to factor each ICD-10 code as a 7404-dimensional dummy variable. This simple encoding scheme might, however, be improved upon, typically by exploiting the ICD-10 hierarchical structure by considering codes as sequences of character. This approach was investigated, but yielded significantly lower results. As a consequence, the results reported in this article only concern the dummy variable encoding scheme.

### Miscellaneous Variables

From gender to birth town, a death certificate contains various additional information items on its subject besides the chain of events leading to death. As some of these items are typically used by both Iris and human coders to decide the underlying cause of death, they present an interest as explanatory variables for the investigated predictive model. After consultation with expert coders, the following items available on French death certificates were selected as additional exogenous variables:

1. Gender: two states of categorical variables.
2. Year of death: 16 states of categorical variables.
3. Age, factorized into 5-year intervals from subjects less than 1 year old, which were divided into two classes.

### Neural Architecture

With the death certificate and its selected variables converted into a format enabling analysis, the underlying cause of death extraction task can be solved by estimating its corresponding ICD-10 code’s probability density, conditioned on the explanatory variables defined previously:

*P*(*UCD*|*CCD*,*A*,*Y*,*G*,*Θ*) = *ƒ_Θ_*(*CCD*,*A*,*Y*,*G*) (2)

with *UCD* ε R^7404^ representing the underlying cause of death, *CCD* ε R^6^ × R^20^ × R^7404^ representing the ICD-10 grid−encoded causal chain of death, *A* ε R^25^ representing the categorized age, *Y* ε R^16^ representing the year of death, *G* ε R^2^ representing the gender, and *ƒ_Θ_* representing a mapping from the problem’s input space to its output space, parameterized in *Θ*, a real-valued vector, typically a neural network.

**Figure 3:**
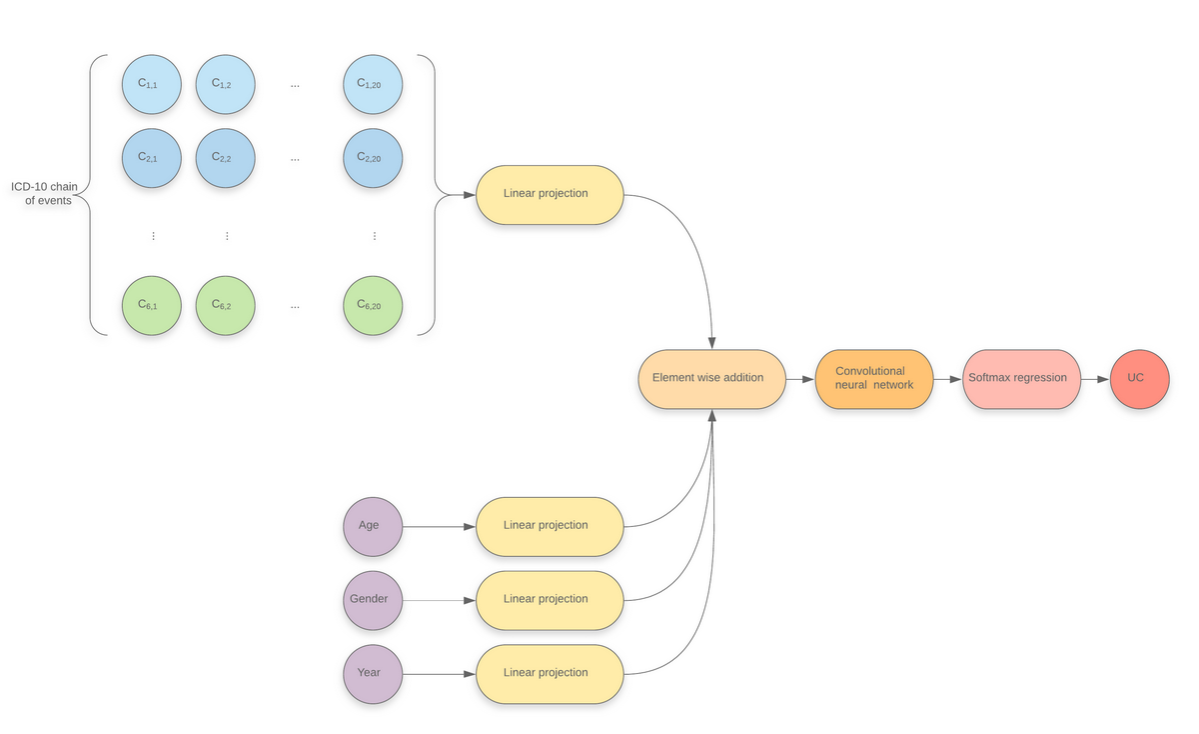
Overall model architecture. Ci,j: denotes the jth code at the ith line in the death certificate; ICD-10: 10th revision of the International Statistical Classification of Diseases and Related Health Problems; UC: underlying cause.

Although properly defined, the investigated prediction problem still presents significant challenges for traditional statistical modelling methods. First, it is expected that the relationship between the input variables and the investigated regressand should be highly nonlinear, whereas most statistical modelling techniques are typically used in linear settings. Feed-forward neural networks [15], however, were developed as powerful nonlinear expansions of traditional linear or logistic regressions with state-of-the-art performance in a wide variety of tasks, typically in computer vision and natural language processing. Although the currently investigated modelling problem does not fall into one of these categories, recent advances in both deeply inspired the neural architecture presented in this article, which can be seen in Figure 3 and can be decomposed as follows:

1. Linear projections are applied to each one-hot encoded categorical variable [16] (ie, one linear projection is shared for all ICD-10 codes present in the causal chain of death), with all linear projections sharing the same output space dimension.
2. The miscellaneous variables’ projections are added to all of the projected grid’s elements.
3. The resulting grid is used as input to a convolutional neural network [17].
4. A multinomial logistic regression (ie, softmax regression) targeting the underlying cause of death is performed on the convolutional neural network’s output [18].
5. All model parameters (ie, from both the linear projections and the convolutional network) are adjusted by minimizing a cross-entropy objective using gradient-based optimization. The model’s gradients are computed using the backpropagation method [15].

The authors feel that the formal definition of all the model’s constituents fall outside the scope of this article. The interested reader will, however, find a complete description of the model in Multimedia Appendix 1, as well as a fully implemented example, written with Python and TensorFlow, in Falissard [19]. We also encourage interested readers to explore the multiple articles that influenced this architecture’s design, which are all available in the bibliography [16,20-22].

### Training and Evaluation Methodology

The investigated model was trained using all French death certificates from 2000 to 2015. A total of 10,000 certificates were randomly excluded from each year and spread into a validation set for hyper-parameter fine-tuning, and a test dataset for unbiased prediction performance estimation (5000 each), resulting in three datasets with the following sample sizes:

1. Training dataset: 8,553,705 records.
2. Validation and test dataset: 80,000 records each.

Being approximately 1% of the training set’s size, the validation and test sets might appear unreasonably small. This is, however, standard practice in the machine learning academic literature when handling big datasets (ie, several millions of training examples) [23]. In addition, the final model shows the same performances on the validation and test sets—up to a tenth of a percent—thus constituting strong evidence as to the sample distribution’s stability.

The model was implemented with TensorFlow [24], a Python-based distributed machine learning framework, on two NVIDIA RTX 2070 GPUs (graphics processing units) simultaneously using a mirrored distribution strategy. Training was performed using a variant of stochastic gradient descent, the Adam optimization algorithm.

The numerous hyper-parameters involved in the model and optimization process definition were tuned using a random search process. However, due to the significant amount of time required to reach convergence on the different versions of the model trained for the experiment (ie, around 1 week per model), only three models were trained, the results displayed below being reported from the best of them, in terms of prediction accuracy on the validation set. The interested reader will find a complete list of the hyper-parameters defining this model in Multimedia Appendix 1 (see Table MA1-1). Given the considerably small hyper-parameter exploration performed for the experiment reported in this article, the authors expect that better settings might provide with a slight increase in prediction performance. However, given the successful results obtained and the computational cost of a finer tuning, a decision was taken to not further the exploration.

After training, the model’s predictive performance was assessed on the test dataset, which was excluded prior to training as mentioned earlier, and compared to that of the Iris software, nowadays considered as the state-of-the-art in automated coding and internationally used. In order to ensure a fair comparison between the two systems, Iris’ performances were assessed on the test set as well and given the same explanatory variables. As is done traditionally in the machine learning academic literature, the predictive performance is reported in terms of prediction accuracy, namely the fraction of correctly predicted codes in the entire test dataset.

The Iris software’s automatic coding accuracy was assessed with two distinct values resulting from the software’s ability to automatically reject cases considered as too complex to be handled by the decision system. As a consequence, a first accuracy measurement—the lowest one—was assessed considering rejects as ill-predicted cases, while the second one excluded these rejects from the accuracy computation, thus yielding an improved estimate. In order to present the reader with a more comprehensive view of both approaches’ performances, these accuracy metrics were also derived on a per-chapter basis, again on the same test set.

## Results

### Overview

The neural network−based model was trained as described previously for approximately 5 days and 18 hours, and its predictive performance as well as that of Iris are reported in Table 1.

The neural network−based approach to the automated coding of underlying cause of death significantly outperforms the state-of-the-art regarding both metrics. Indeed, even when compared to Iris’ performance on nonrejected cases, the error rate offered by the proposed approach is 3.4 times lower. This performance difference increases to an 11-fold decrease when including rejected cases in Iris performance.

**Table 1:** Prediction accuracy of Iris and the best derived predictive model derived by bootstrap.

Selected approach	Prediction accuracy	95% CI
Iris overall accuracy	0.745	0.740-0.750
Iris on nonrejected certificates	0.925	0.921-0.928
Proposed approach	0.978	0.977-0.979

**Figure 4:**
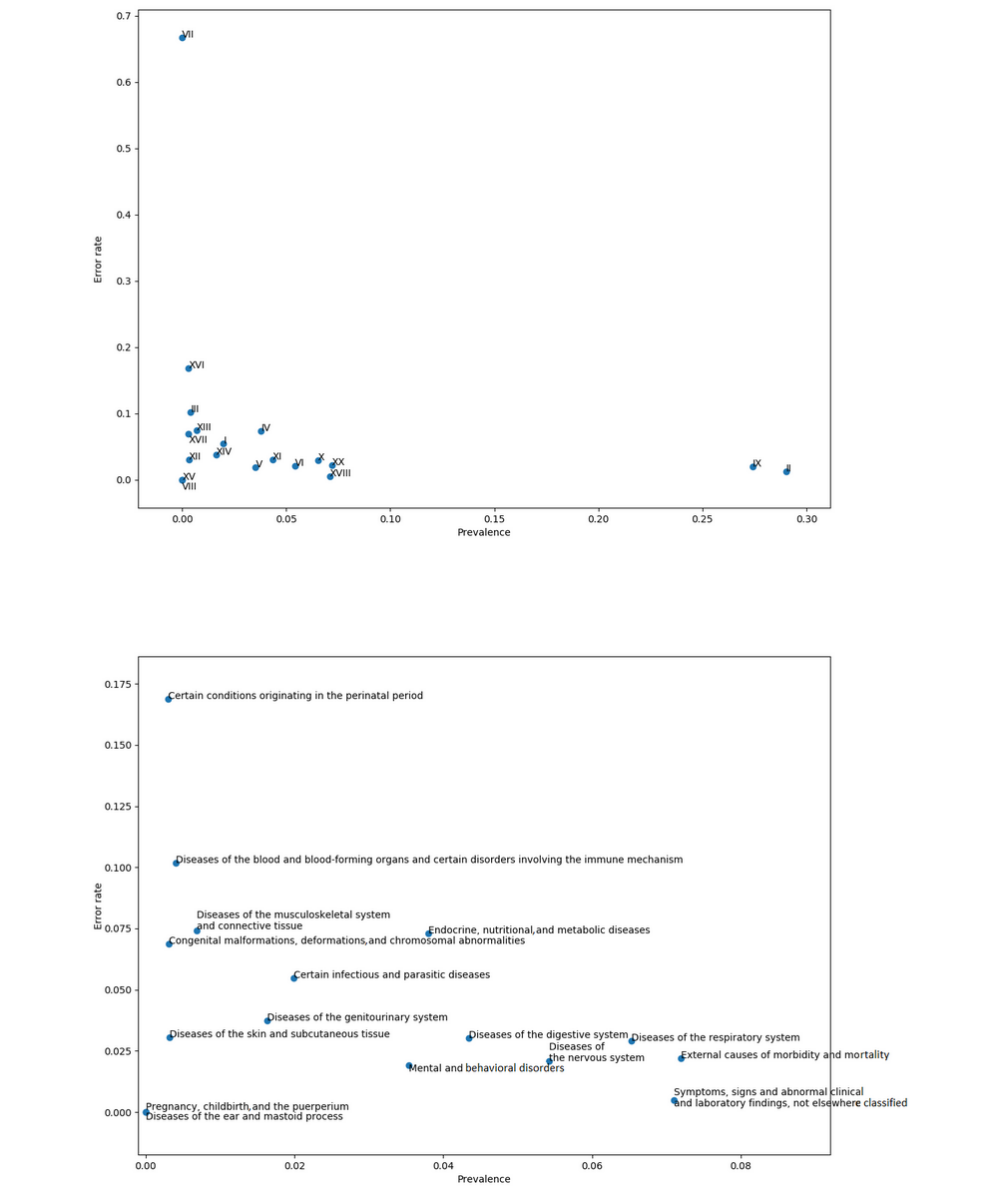
The top plot shows the relevance of underlying causes (by ICD-10 chapter) against ICD-10 chapter−level model error rate. The bottom plot is a zoom on the top plot’s bottom left-hand corner. ICD-10: 10th revision of the International Statistical Classification of Diseases and Related Health Problems.

In addition, Figure 4 shows the model’s error rates per ICD-10 chapter, alongside the latter’s prevalence. In this plot, chapter VII—diseases of the eye and adnexa—appears as a strong outlier in terms of error rate. Although not statistically significant (ie, only 3 death certificates among the 80 thousands sampled for the test set have a chapter VII−related underlying cause of death), this observation might indicate that the training set does not have a big enough sample size to allow the model to handle extremely rare cases such as chapter VII−related death certificates, which might better be handled by a hand-crafted, rule-based decision system.

**Figure 5:**
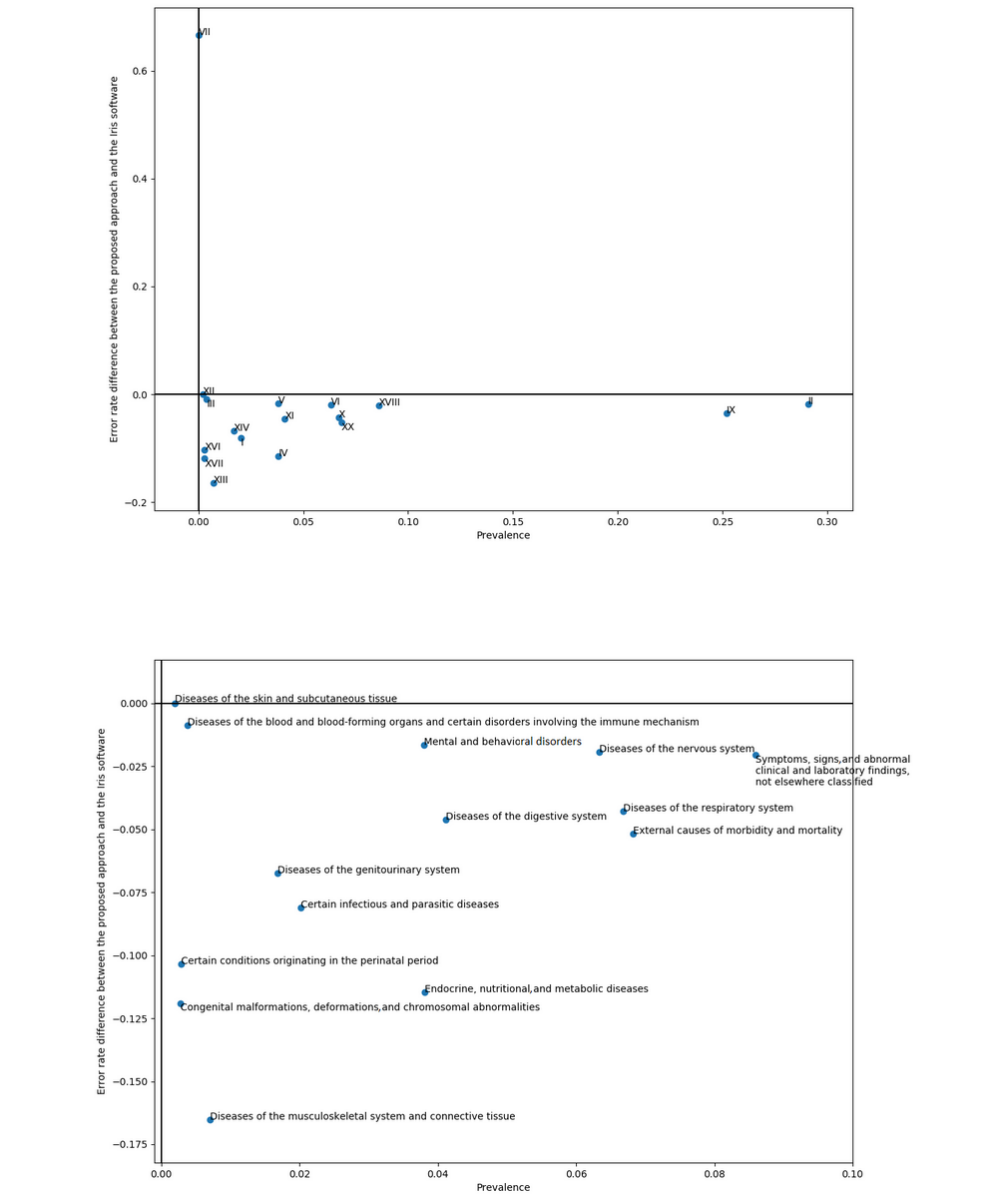
The top plot shows the difference in error rate between the proposed model and the Iris software versus ICD-10−chapter prevalence as underlying cause. The bottom plot is a zoom on the top plot’s bottom left-hand corner. ICD-10: 10th revision of the International Statistical Classification of Diseases and Related Health Problems.

Finally, Figure 5 shows the per-chapter difference in error rate between the proposed neural network approach and the Iris software, on nonrejected certificates. As previously hypothesized, the Iris software outperforms the deep learning approach on diseases of the eyes and adnexa-related death certificates (chapter VII), although still not significantly. Even if the Iris software is beaten in every other chapter, a case should be made from never-appearing chapters. Indeed, a number of chapters—namely, chapters XIX, XXI, and XXII—are not observed as underlying causes in the test dataset, strongly indicating that they might benefit from a set of hand-crafted rules, as do chapter VII−related certificates, if they were to appear in extremely rare cases.

### Error Analysis

Although the proposed approach significantly outperforms the current state-of-the-art that is the Iris software, neural network−based methods are known to present several drawbacks that can significantly limit their application in some situations. Typically, the current lack of systematic methods to interpret and understand neural network−based models and their decision processes can lead the former to perform catastrophically on ill-predicted cases, independently from their high predictive performances.

As a consequence, the proposed model behavior in ill-predicted cases requires careful analysis. In addition, the system’s performance can potentially benefit from such an investigation. For instance, although the model outperforms Iris on average, there might some highly nonlinear exceptions that are better fit to rule-based decision systems, in which case a hybrid approach could, by using the best of both worlds, again yield performance gains.

**Figure 6:**
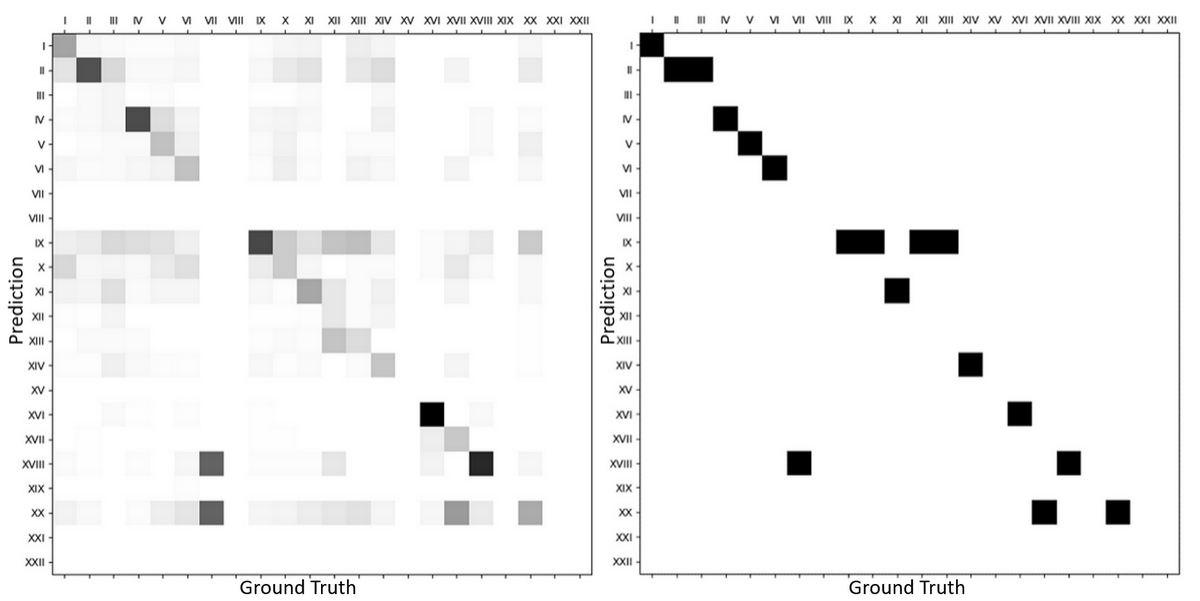
The left-hand plot shows the distribution of wrong predictions per ICD-10 chapter versus their ground truth (the lighter the rarer). The right-hand plot shows the same distribution’s modes. Apparent missing values in both plots correspond to chapters either not represented in the test dataset or on which no mistakes were made. ICD-10: 10th revision of the International Statistical Classification of Diseases and Related Health Problems.

Although assessing per-chapter error rates, as previously shown, constitutes a simple, straightforward approach to understanding the model’s weakness, much more can be done to gain insight into the model’s behavior. As an example, it only feels natural, after identifying cases incorrectly predicted by the investigated model, to assess the nature of errors made by the latter. As aforementioned, neural network−based classifiers tend to, in misprediction cases, output answers unreasonably far from the ground truth. One should, however, expect from a good predictive model to, in error cases, output predictions as close as possible to the correct answer. Figure 6 displays an ICD-10 chapter−level confusion matrix built from ill-predicted test cases, and shows that, besides chapter VII, most of the errors remain in the same chapter as the ground truth, indicating some degree of model robustness.

**Figure 7:**
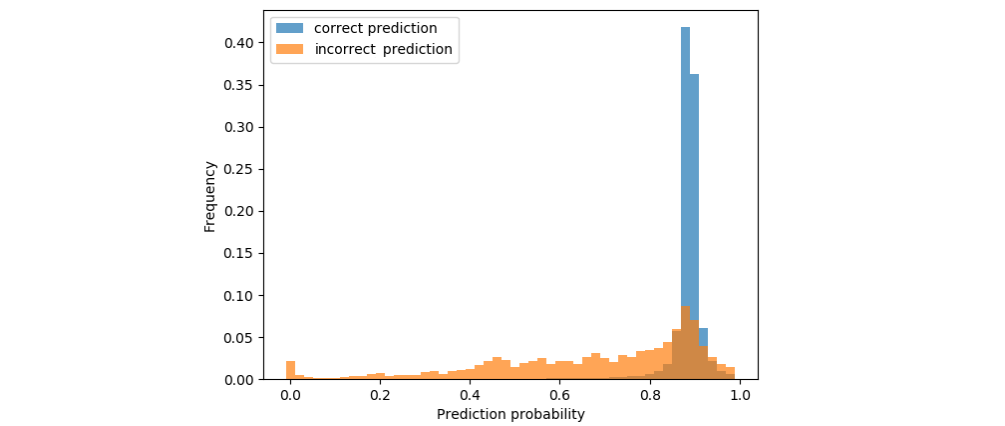
Prediction confidences are shown in correct (blue) and incorrect (orange) predictions. The model typically predicts correct values with high confidence and incorrect values with lower confidence.

The model’s error behavior can also be investigated from a calibration fitness perspective. As aforementioned, some artificial neural network−based models have been known to behave quite poorly in ill-predicted cases, which could constitute a highly undesirable phenomenon when handling health data. When the model is being fit in a similar fashion to multinomial logistic regression, it does not directly learn to predict an ICD-10 code, but instead estimates a discrete conditional probability distribution across all possible codes. The prediction, defined as the argument of the maxima on said distribution, is consequently associated with a probability weight that, when properly calibrated, can be considered as a confidence score on individual model predictions. Typically, a well-calibrated predictive model would be expected to show high confidence in cases where the prediction is correct, and a low one when mispredicting. Bar plots of said prediction confidences can be found in Figure 7 and clearly show a strong tendency for the model to be more confident in its prediction in correctly predicted cases.

If predicting incorrect values with low confidence is a desirable behavior for a predictive model, associating the ground truth with high probabilities, even in misprediction cases, should be of equal importance. This is typically assessed by evaluating whether each test set subject’s corresponding ground truth is contained in the *k* ε N* most probable values present in the model’s corresponding outputted distribution. This type of metric is typically denoted as the model’s top-*k* accuracy, and helps in assessing a model’s ability to give high confidence to correct values, even when mispredicting. Although the academic machine learning literature typically makes use of the top-5 accuracy in such cases, the investigated model was investigated with a top-2 accuracy only. Indeed, most death certificates present in the dataset display causal chains of events with five or less ICD-10 codes, with the underlying cause of death being one of them. It is consequently reasonable to expect the model to output these five codes as most probable, thus leading to a high but meaningless top-5 accuracy. The assessed top-2 accuracy can be found in Table 2, and strongly indicates that the model consistently associates correct underlying causes of death with higher probabilities, even in ill-predicted cases.

**Table 2:** Accuracies on codes wrongly predicted by the proposed model, and the model’s top-2 accuracy.

Performance metric	Value	95% CI
Second-most probable code prediction accuracy on ill-predicted certificates	0.663	0.641-0.685
Proposed model’s top-2 accuracy	0.993	0.992-0.993

A richer, although more time-consuming, error analysis can be derived from human observation of each error case by an underlying cause of death coding specialist. To do so, 96 of the 1777 ill-predicted death certificates in the test set were selected at random and shown to the medical practitioner referent and final decision maker on underlying cause of death coding in France, who gave the following for each of the selected certificates:

1. Her personal opinion of what each certificate’s corresponding underlying cause should be.
2. A qualitative comment on the investigated model’s error.

The aforementioned underlying causes obtained were then confronted with both the actual values contained in the dataset and those predicted by the derived model, leading to the following observations:

1. In 41% (39/96) of cases, the referent agreed with the model’s predictions.
2. In 38% (36/96) of cases, the referent agreed with the underlying cause present in the dataset.
3. In 22% (21/96) of cases, the referent disagreed with both of them.

From these certificates, 4 were randomly selected where the medical referent disagreed with the proposed predictive model, and these are displayed in Multimedia Appendix 1. These errors can be grouped into three distinct categories:

1. Certificates displayed in Tables MA1-2 and MA1-3 are mistakes depending on highly nonlinear, almost casuistic rules and are typical examples of scenarios where a hybridized deep learning- and expert-based system should be beneficial.
2. The certificate displayed in Table MA1-4 constitutes a rare, complex death scenario that would require the expertise of a medical referent.
3. The certificate displayed in Table MA1-5 is compatible with several underlying causes of death, and the underlying cause of death ICD-10 code’s fourth character is left at the coder’s discretion.

It appears from this experiment that the derived predictive model’s coding can be considered as comparable in quality to the actual process responsible for the production of that of the investigated dataset. In addition, a qualitative analysis of the medical practitioner’s comments on the model’s mistakes showed that 30% of errors committed by the predictive model are related to casuistic exceptions in coding rules, such as nonacceptable codes as underlying causes of death. Such an observation strongly reinforces the hypothesis that a hybrid expert system–deep learning approach should improve the presented system’s coding accuracy.

### Availability of Data and Materials

The data that support the findings of this study are available from the French Epidemiological Centre for the Medical Causes of Death, but restrictions apply to the availability of these data, which were used under license for this study, and so are not publicly available. Data are, however, available from the French Epidemiological Centre for the Medical Causes of Death upon reasonable request.

## Discussion

### Principal Findings

The results of the previous handmade error analysis raise some questions regarding the underlying cause of death coded in the training dataset’s quality, as well as its impact on the proposed predictive model. Indeed, both the Iris software and the human coders are not exempt from making mistakes, thus making the underlying cause of death ground truth not entirely reliable. Investigation of human coder performances have already been conducted and reported intercoder and intracoder agreements as low as 70% and 89%, respectively, on more complex cases [25]. These scores can, at least partially, be explained by the subtle differences sometimes existing between codes denoting similar pathologies. The ICD-10’s granularity can sometimes render the underlying cause of death decision process slightly stochastic for human coders. A well-known example of this phenomenon can be seen in the previously shown error example, with diabetes-related deaths. However, measurement noise has always been an ubiquitous part of medical datasets, and expecting a perfect, deterministic coding process based on human decisions seems somewhat unreasonable. In addition, statistical predictive models, which deep learning models are, have been known to perform relatively well when confronted with noisy datasets. Finally, the model’s substantial predictive performances make a strong argument toward the ground truth underlying cause of death’s coding quality.

Finally, the necessity of including the miscellaneous variables in the model should be thoroughly assessed. Indeed, although these variables are usually available in a straightforward fashion on death certificates, minimizing the amount of additional information given to the model is a topic of importance. The year and age variables both have an a priori known, deterministic effect on the coding process.

The age variable explicitly intervenes in some WHO-defined rules. As an example, neonatal deaths (<28 days) are subject to an entirely different set of both ICD-10 codes and rules [8]. As a consequence, excluding any information on the subject’s age from the model would deterministically impair its predictive performances.

Strictly speaking, the subject’s year of passing should only have a limited effect on the underlying cause of death. However, the WHO-defined coding rules are subject to changes over the years, from the addition of new ICD-10 codes to changes in the decision processes themselves [26]. As a consequence, the model should benefit, in terms of predictive performance, from being able to differentiate between different years. In addition, including this variable in the model would allow practitioners to recode entire parts of the dataset with rules learned from a given year, thus smoothing temporal distribution variabilities.

The gender variable, however, does not appear to influence any coding rules, but was added following the French cause of death coding medical expert’s opinion. In order to assess its interest in the investigated decision process, an ablation study was realized. The proposed model was trained with the gender variable excluded, leading to no significant change in prediction performance, strongly supporting the thesis that the gender information does not influence the decision process and should not be included in future related works.

### Practical Application: Recoding the 2012 French Overdose Anomaly

The topic of overdose-related death monitoring has recently drawn the attention of public health agencies around the world, specifically in light of the opioid-related sanitary crisis recently witnessed in the United States. Causes-of-death data constitute an information source of choice to investigate such topics. In France, the CépiDc database was used to assess the evolution of overdose-related deaths from 2000 to 2015, by counting, for each year, the number of deaths associated with the following underlying causes (ICD-10 codes shown in parentheses):

1. Opioid- and cannabis-related disorders (ICD-10 codes beginning with F11 and F12).
2. Cocaine-, hallucinogen-, and other stimulant-related disorders (F14 to F16).
3. Other psychoactive substance−related disorders (F19).
4. Accidental poisoning by, and exposure to, narcotics and psychodysleptics, not elsewhere classified (X42).
5. Intentional self-poisoning by, and exposure to, narcotics and psychodysleptics, not elsewhere classified (X62).
6. Poisoning by, and exposure to, narcotics and psychodysleptics, not elsewhere classified, with undetermined intent (Y12).

**Figure 8:**
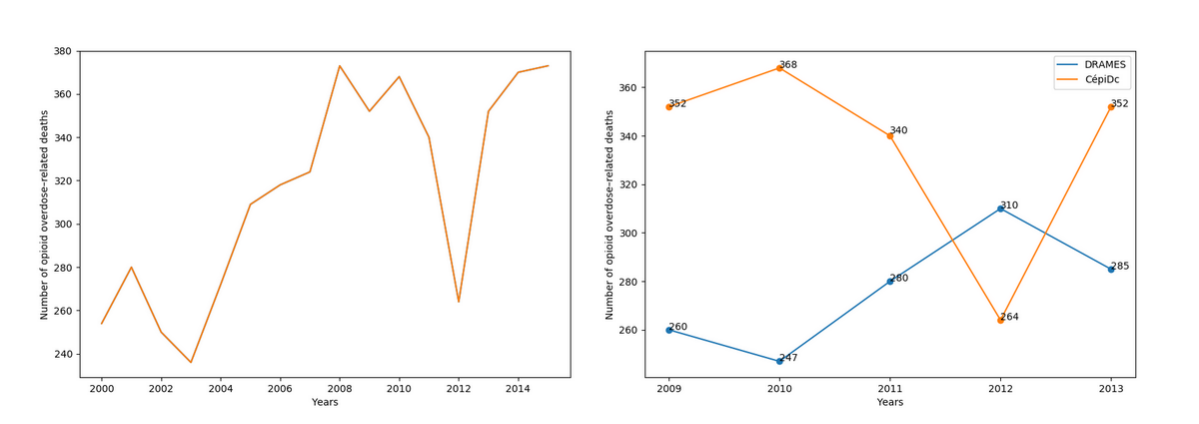
The left-hand plot shows the evolution of overdose-related deaths from 2000 to 2015 in France. The sudden decrease in 2012 appears anomalous. The right-hand plot shows the comparison with DRAMES (Décès en Relation avec l’Abus de Médicaments Et de Substances) data, a nonexhaustive, independent data source, which finds more deaths in 2012 than the exhaustive CépiDc (Centre d’épidémiologie sur les causes médicales de Décès) database.

The resulting trajectory can be found in Figure 8 and shows a significant decline in overdose-related deaths in 2011 and 2012.

Although this punctual reduction can be at least partially explained by observed decreases in both heroin purity [27] and heroin overdose−related deaths [28] in the same time period, confrontation with results obtained from an independent source, the DRAMES (Décès en Relation avec l’Abus de Médicaments Et de Substances) dataset, suggests another hypothesis. The DRAMES study constitutes a nonexhaustive inventory of overdose-related deaths detected in French Legal Medicine Institutes. As a nonexhaustive database, its death count should not exceed the value obtained from the CépiDc database. As can be seen in Figure 8, this logical assertion is true for all years from 2009 to 2013, with a notable exception of 2012. This discrepancy might be explained by a coding process deficiency, a hypothesis that can easily be verified by recoding every certificate from 2012 and comparing the number of overdose-related deaths in both situations.

The model derived in the previous experiment was used to recode every French death certificate from 2000 to 2015, with the year of coding set to 2015 to prevent any discrepancy related to coding rule variation. The overdose-related deaths were then selected from the predicted underlying causes of death following the aforementioned methodology.

**Figure 9:**
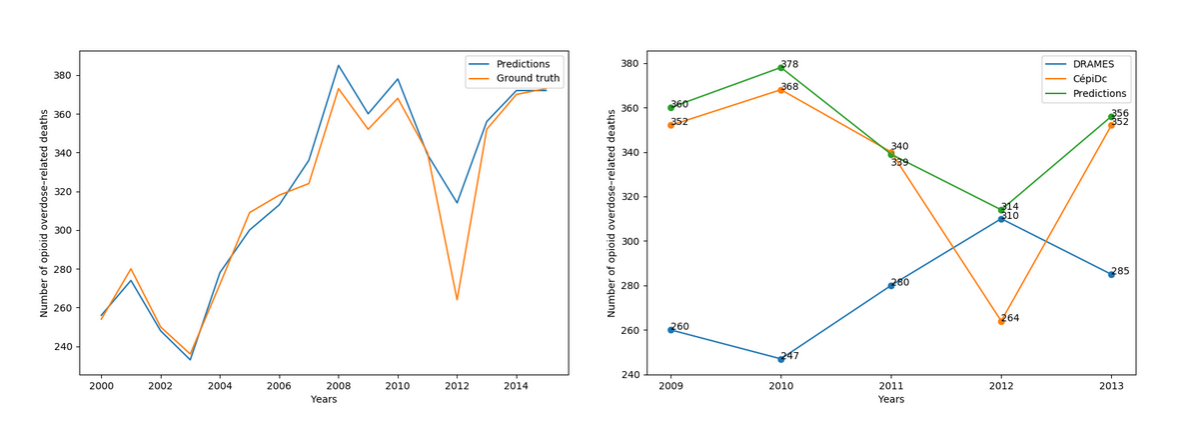
The left-hand plot shows the evolution of opioid overdose−related deaths from 2000 to 2015 in France, either coded with Iris and human coders (orange) or with the proposed approach (blue). The 2012 gap, although still present, is much smoother when using predicted underlying causes. The right-hand plot shows the comparison with DRAMES (Décès en Relation avec l’Abus de Médicaments Et de Substances) data. The contradiction with the CépiDc (Centre d’épidémiologie sur les causes médicales de Décès) database is entirely corrected with the predicted causes.

The resulting curve can be seen in Figure 9, alongside the official curve, and clearly shows a smoother decrease in opioid-related deaths. The discrepancy with the DRAMES database, in addition, disappears when considering the recoded underlying causes of deaths.

### Conclusions

In this article, we presented a formulation of the underlying cause of death coding from death certificates as a statistical modelling problem, which was then addressed with a deep artificial neural network, setting a new state-of-the-art. The derived model’s behavior was thoroughly assessed following different approaches in order to identify potentially harmful biases and assess the potential of a hybrid approach mixing a rule-based decision system and statistical modelling. Although the proposed solution significantly outperformed any other existing automated coding approaches on French death certificates, the question of model transferability to other countries requires more investigation. Indeed, the problem of distribution shift is well known in the machine learning community and can significantly impair the model’s quality [29].

The authors feel confident that the model should perform with similar predictive power on other countries’ death certificates with little to no supplementary effort necessary, even though this claim requires some experimental validation, unrealizable without international cooperation. To conclude, this article shows that deep artificial neural networks are perfectly suited to the analysis of electronic health records and can learn a complex set of medical rules directly from voluminous datasets, without any explicit prior knowledge. Although not entirely free from mistakes, the derived algorithm constitutes a powerful decision-making tool able to handle structured, medical data with unprecedented performance. We strongly believe that the methods developed in this article are highly reusable in a variety of settings related to epidemiology, biostatistics, and the medical sciences in general.

## Appendix

Multimedia Appendix 1 Model architecture, training methodology, and examples of mispredicted certificates.

## Abbreviations

CépiDc: Centre d’épidémiologie sur les causes médicales de Décès
DRAMES: Décès en Relation avec l’Abus de Médicaments Et de Substances
GPU: graphics processing unit
ICD-10: 10th revision of the International Statistical Classification of Diseases and Related Health Problems
WHO: World Health Organization
