# Supplementary material for: A Deep Artificial Neural Network−Based Model for Prediction of Underlying Cause of Death From Death Certificates: Algorithm Development and Validation
Source: JMIR Med Inform. 2020 Apr 28;8(4):e17125. doi: 10.2196/17125 (PMC7218605; doi:10.2196/17125)
Supplement: Multimedia Appendix 1 [file medinform_v8i4e17125_app1.docx]

A deep artificial neural network based model for underlying cause of death prediction from death certificates, Annex

# Model architecture

The model architecture is mostly inspired from the Inception v2 network (1), with the following modifications:

- The Inception v2 network’s first stage has been replaced with two temporal blocks (2) with successive dilation rates of 1 and 2
- Drop-out (3) , layer normalization (4) and residual connections (5) were applied to each block as can be seen on Figures MA1-1, MA1-2 and MA1-3
- The Inception maximum pooling operation was limited to the grid’s width dimension as can be seen on Figure MA1-4
- The final softmax operation is tied to the linear embedding as described in (6)

The model’s full structure is described in Table MA1-1 and Figures MA1-1 through MA1-4.

| Type | Layer size | Dilation rate, stride or remarks | Input size |
| --- | --- | --- | --- |
| Linear embedding | $512$ | $\_$ | $6\times20\times7404$ |
| Temporal block (2) | $512$ | $1$ | $6 \times20 \times512$ |
| Temporal block | $512$ | $2$ | $6 \times20 \times512$ |
| 3 x Inception block 1 (1) | $512$ | As in Figure MA1-1 | $6 \times20 \times512$ |
| Inception pooling (1) | $1024$ | As in Figure MA1-4 | $6\times20 \times512$ |
| 5 x Inception block 2 (1) | $1024$ | As in Figure MA1-2 | $6 \times9 \times1024$ |
| Inception pooling | $1536$ | As in Figure MA1-4 | $6 \times9 \times1024$ |
| 2 x Inception block 3 (1) | $1536$ | As in Figure MA1-3 | $6 \times4 \times1536$ |
| Full maximum pooling | $1536$ | $6 \times4$ | $6 \times4 \times1536$ |
| Linear layer | $512$ | $\_$ | $1 \times1 \times1536$ |
| Tied linear embedding (6) | $7404$ | Transpose of the first linear embedding matrix | $1 \times1 \times512$ |

Table MA1-1: Model architecture and corresponding hyperparameters


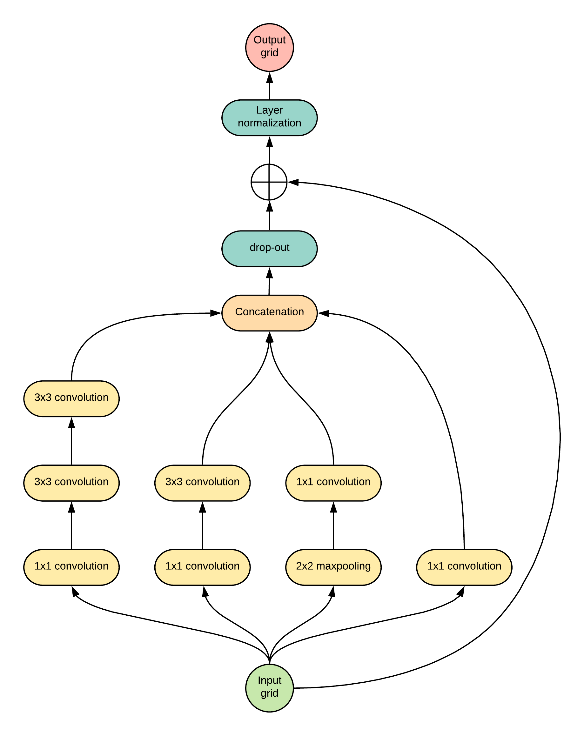


Figure MA1-1: Inception block 1 as described in (1) with additional drop-out, layer normalization and residual connection mechanisms


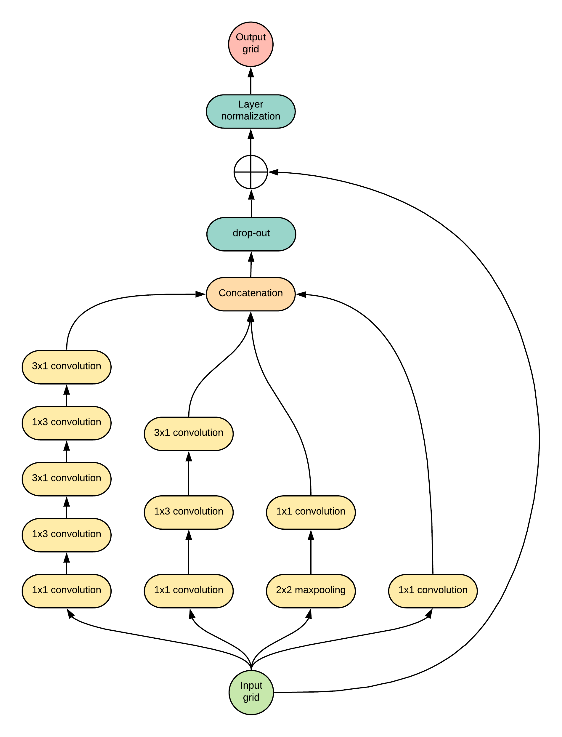


Figure MA1-2: Inception block 2 as described in (1) with additional drop-out, layer normalization and residual connection mechanisms


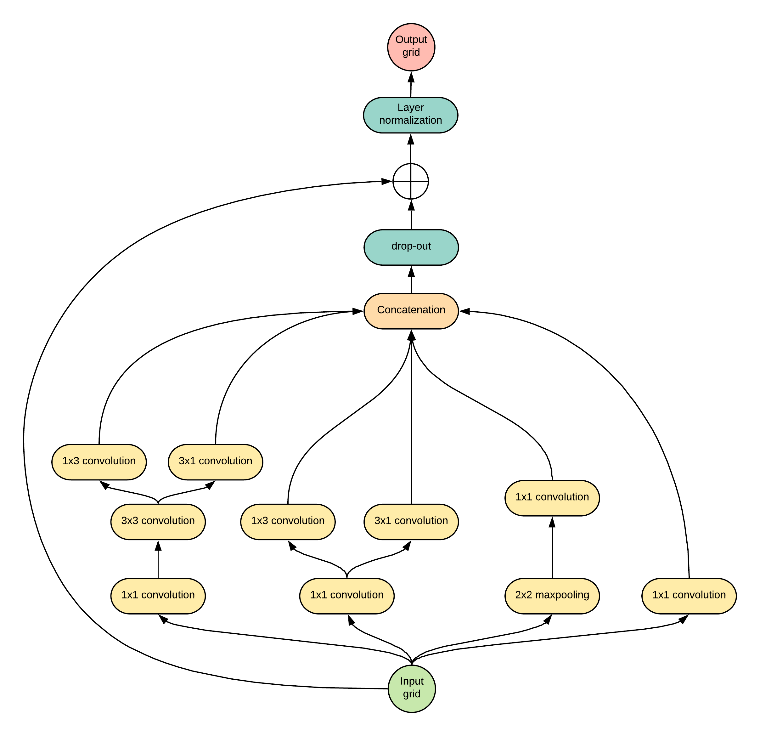


Figure MA1-3: Inception block 3 as described in (1) with additional drop-out, layer normalization and residual connection mechanisms


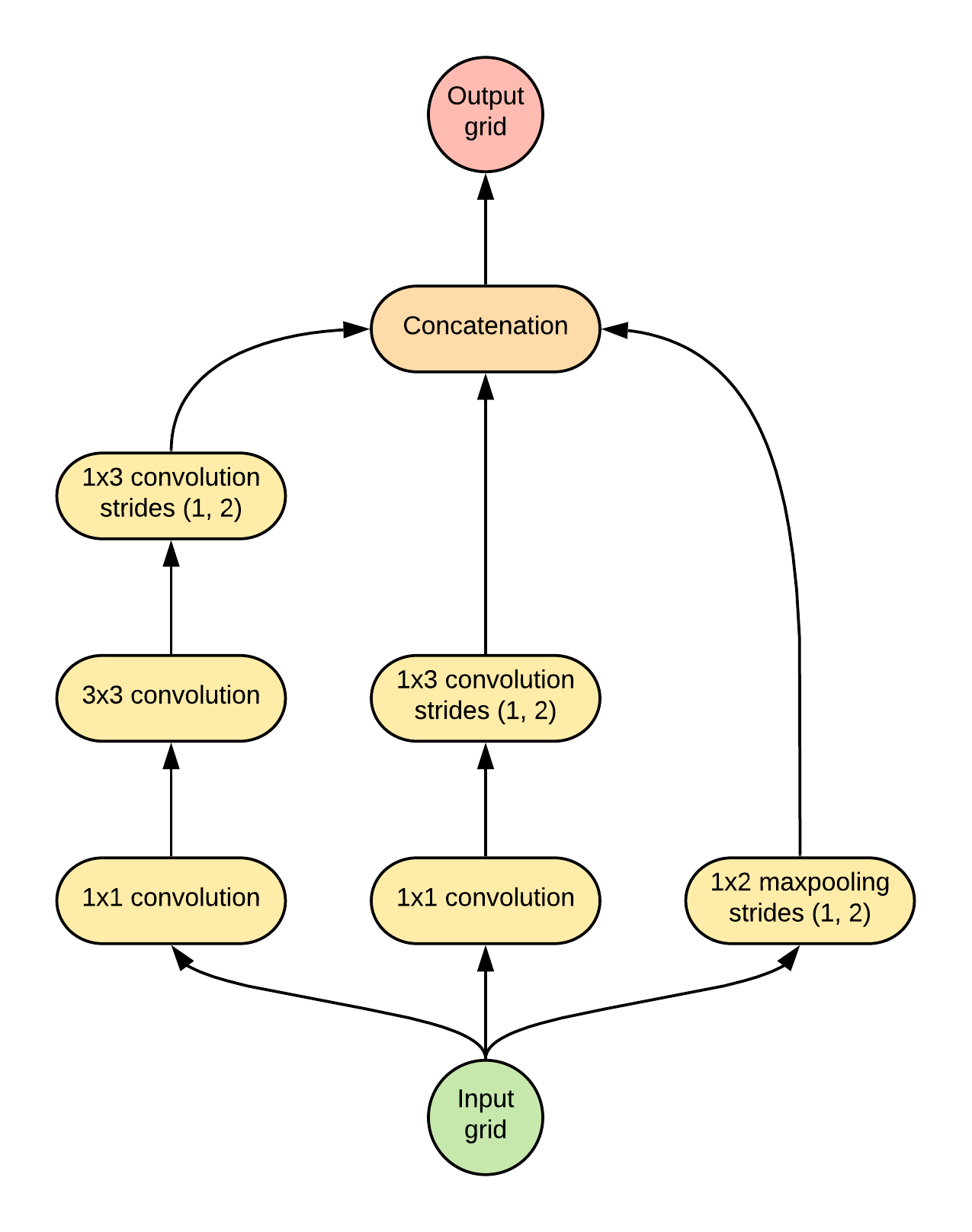


Figure MA1-4: Inception pooling as described in (1). All grid-reducing operations are limited to the width dimension

# Training methodology

The model was implemented with Tensorflow, a python-based distributed machine learning framework, on two NVidia RTX 2070 GPUs simultaneously using a mirrored distribution strategy. Training was performed using a variant of stochastic gradient descent, the Adam optimization algorithm.

The descent’s step size (also called learning rate in the machine learning academic literature) was updated in real time during training according a rule defined in (7), that can be seen in figure X and is defined according to the formula:

$$Step\_size\left( t \right)= \alpha\cdot\min\left( t^{-0.5}, t \cdot{warmup\_steps}^{-1.5} \right) \forall t\in\mathbb{R}^{+}$$

With:

- $\alpha\mathbb{\in R}$ a constant considered as a model hyper-parameter and defining the learning rate’s overall amplitude
- $warmup\_steps\mathbb{\in N}$ another hyper-parameter defining the learning rate’s linear warmup phase length


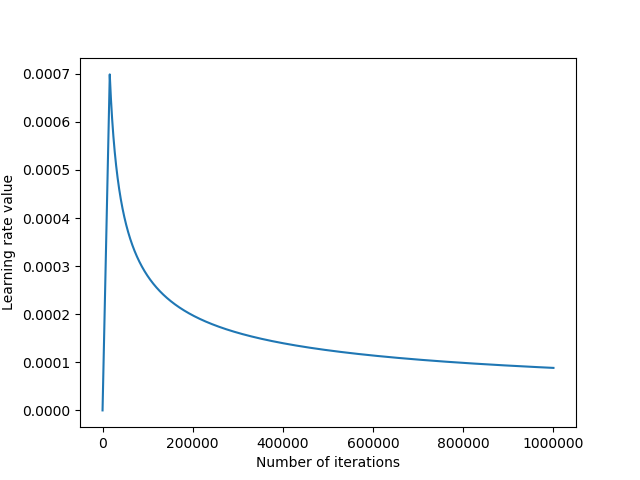


Figure MA1-5 learning rate evolution with gradient descent iterations. The learning rate follows a first linear increase warmup phase followed by an inverse root square decay

In order to limit gradient explosion phenomena typically encountered in deep neural network, the optimization was in addition controlled using gradient clipping. Essentially, the norm of all gradients computed during the descent were normalized to be of global norm equal or less than 0.1.

In addition, label smoothing was applied to the cross entropy loss to further regularize the model.

The final hyper parameters were chosen from a random search selection process with the following values:

- Batch size: 250
- Drop-out selection rate: 0.1 for all layers
- Label smoothing parameter: 0.1
- Initial learning rate constant: $\frac{2}{\sqrt{512}}\approx0.088$
- Learning rate warmup steps: 16000
- Trainable variable initialization: Uniform variance scaling initializing

# Example of mispredicted certificates

| Line | Content | | |
| --- | --- | --- | --- |
| 1 | I501 Left ventricular failure | | |
| 2 | J690 Acute respiratory failure | | |
| 6 | I509 Heart failure, unspecified | | |
|  | | | |
| Source | Medical expert | Prediction | Dataset |
| UCD | I501 | J690 | I501 |

Table MA1-2 Example of death certificate where the prediction differs from the underlying cause of death presented in the dataset. The medical expert agreed with the code present in the dataset, and commented that J690 is not a valid underlying cause of death code

| Line | Content | | |
| --- | --- | --- | --- |
| 1 | R688 Other specified general symptoms and signs | | |
| 2 | N19 Unspecified kidney failure E148 Unspecified diabetes mellitus with unspecified complications | | |
| 3 | I499 Cardiac arrhythmia, unspecified I519 Heart disease, unspecified I501 Left ventricular failure E46 Unspecific protein-energy malnutrition | | |
| 4 | Z896 Acquired absence of leg above knee | | |
| 5 | I702 Atherosclerosis of arteries of extremities | | |
| 6 | Z740 Need for assistance due to reduced mobility I694 Sequelae of stroke, not specified as haemorrhage or infarction | | |
|  | | | |
| Source | Medical expert | Prediction | Dataset |
| UCD | I501 | I702 | I501 |

Table MA1-3 Example of death certificate where the prediction differs from the underlying cause of death presented in the dataset. The medical expert agreed with the code present in the dataset, and commented that this certificate is subject to “linked causes” a set of casuistic exceptions

| Line | Content | | |
| --- | --- | --- | --- |
| 1 | Q300 Choanal atresia | | |
| 2 | Q878 Other congenital malformation syndromes, not elsewhere classified | | |
| 3 | Q213 Tetralogy of Fallot Q165 Congenital malformation of inner ear I678 Other specified cerebrovascular diseases | | |
| 6 | P013 Fetus and newborn affected by polyhydramnios | | |
|  | | | |
| Source | Medical expert | Prediction | Dataset |
| UCD | Q897 Multiple congenital malformations,  not elsewhere classified | Q878 | Q300 |

Table MA1-4 Example of death certificate where the prediction differs from the underlying cause of death presented in the dataset. The medical expert disagreed with both values, and commented that this certificate constitutes a “rare case requiring the medical referent’s expertise”

| Line | Content | | |
| --- | --- | --- | --- |
| 1 | I509 Heart failure, unspecified | | |
| 2 | I259 Chronic ischaemic heart disease, unspecified | | |
| 3 | E109 Type 1 Diabetes mellitus without complications | | |
|  | | | |
| Source | Medical expert | Prediction | Dataset |
| UCD | E108 Type 1 diabetes mellitus with unspecified complications | E106 Type 1 diabetes mellitus with other specified complications | E109 |

Table MA1-5 Example of death certificate where the prediction differs from the underlying cause of death presented in the dataset. The medical expert disagreed with both values, and commented that, when coding diabetes related certificates, the underlying cause of death’s fourth character is often subject to interpretation

# Bibliography

1. Szegedy C, Vanhoucke V, Ioffe S, Shlens J, Wojna Z. Rethinking the Inception Architecture for Computer Vision. arXiv:151200567 [cs] [Internet]. 1 déc 2015 [cité 17 mai 2019]; Disponible sur: http://arxiv.org/abs/1512.00567

2. Bai S, Kolter JZ, Koltun V. An Empirical Evaluation of Generic Convolutional and Recurrent Networks for Sequence Modeling. arXiv:180301271 [cs] [Internet]. 3 mars 2018 [cité 4 févr 2019]; Disponible sur: http://arxiv.org/abs/1803.01271

3. Hinton GE, Srivastava N, Krizhevsky A, Sutskever I, Salakhutdinov RR. Improving neural networks by preventing co-adaptation of feature detectors. arXiv:12070580 [cs] [Internet]. 3 juill 2012 [cité 4 juill 2019]; Disponible sur: http://arxiv.org/abs/1207.0580

4. Ba JL, Kiros JR, Hinton GE. Layer Normalization. arXiv:160706450 [cs, stat] [Internet]. 21 juill 2016 [cité 8 nov 2018]; Disponible sur: http://arxiv.org/abs/1607.06450

5. He K, Zhang X, Ren S, Sun J. Deep Residual Learning for Image Recognition. arXiv:151203385 [cs] [Internet]. 10 déc 2015 [cité 4 juill 2019]; Disponible sur: http://arxiv.org/abs/1512.03385

6. Press O, Wolf L. Using the Output Embedding to Improve Language Models. arXiv:160805859 [cs] [Internet]. 20 août 2016 [cité 17 mai 2019]; Disponible sur: http://arxiv.org/abs/1608.05859

7. [1706.03762] Attention Is All You Need [Internet]. [cité 4 févr 2019]. Disponible sur: https://arxiv.org/abs/1706.03762
